# Genetic variation in lipid desaturases and its impact on the development of human disease

**DOI:** 10.1186/1476-511X-9-63

**Published:** 2010-06-18

**Authors:** Diana M Merino, David WL Ma, David M Mutch

**Affiliations:** 1 University of Guelph, Department of Human Health & Nutritional Sciences, Guelph N1G 2W1, Canada

## Abstract

Perturbations in lipid metabolism characterize many of the chronic diseases currently plaguing our society, such as obesity, diabetes, and cardiovascular disease. Thus interventions that target plasma lipid levels remain a primary goal to manage these diseases. The determinants of plasma lipid levels are multi-factorial, consisting of both genetic and lifestyle components. Recent evidence indicates that fatty acid desaturases have an important role in defining plasma and tissue lipid profiles. This review will highlight the current state-of-knowledge regarding three desaturases (*Scd-1, Fads1 *and *Fads2*) and their potential roles in disease onset and development. Although research in rodent models has provided invaluable insight into the regulation and functions of these desaturases, the extent to which murine research can be translated to humans remains unclear. Evidence emerging from human-based research demonstrates that genetic variation in human desaturase genes affects enzyme activity and, consequently, disease risk factors. Moreover, this genetic variation may have a trans-generational effect via breastfeeding. Therefore inter-individual variation in desaturase function is attributed to both genetic and lifestyle components. As such, population-based research regarding the role of desaturases on disease risk is challenged by this complex gene-lifestyle paradigm. Unravelling the contribution of each component is paramount for understanding the inter-individual variation that exists in plasma lipid profiles, and will provide crucial information to develop personalized strategies to improve health management.

## Introduction

Perturbations in lipid metabolism characterize many of the chronic diseases currently plaguing our society, such as obesity, type 2 diabetes, and cardiovascular disease [1-3]. Lipids constitute a fundamentally important group of diverse metabolites, with critical structural and functional roles within the biological organism. More specifically, many lipid species have been shown to have key roles in such diverse biological processes as signal transduction, membrane trafficking and sorting, morphogenesis, and proliferation [4-6]. While it remains unclear whether perturbations in lipid metabolism are the cause or simply a downstream effect in the development of chronic disease, modifying lipid levels by medical and/or lifestyle interventions remains a primary goal for health management.

Lifestyle factors are typically deemed modifiable risk factors in the development of disease and include high body mass index (BMI), physical inactivity, smoking, alcohol use, and unhealthy eating habits [7-10]. While the authors recognize that each of these lifestyle factors plays an important role in the development of chronic diseases, there is a growing recognition and appreciation of the relationship between diet and health. Indeed the links between the amount and type of dietary fats consumed, and various disease states, are evident in population-based observational studies [11-14]. These studies have associated diets high in saturated fats, refined sugars and high-fat dairy products with a higher incidence of atherosclerosis, cardiovascular disease, metabolic syndrome, cancer and autoimmune diseases. This diet, typically referred to as the Western diet, is commonly associated with a distinct dietary fat composition enriched in saturated fats (SFAs) and n-6 polyunsaturated fatty acids (PUFAs), and poor in n-3 PUFA [15,16]. In contrast a Mediterranean diet emphasizes the consumption of fruits, vegetables, whole grains, wine and poultry, leading to higher intakes of fatty acids such as n-3 PUFAs and monounsaturated fatty acids (MUFAs) [14,17]. These fatty acids are routinely associated with decreased risks for coronary artery disease, hypertension, diabetes, arthritis, inflammatory, and autoimmune diseases [3,8].

Although poor dietary habits can be detrimental to health by themselves, the numerous interactions between nutrients and genes can further modulate an individual's risk for developing disease [18]. The determinants of plasma lipids are multi-factorial; however, it remains unclear to what extent genetic variability contributes to the inter-individual differences observed in plasma lipid profiles. Identifying those gene variants that can modulate lipid levels is crucial for our understanding of the development and severity of disease. While the molecular pathways underlying lipid metabolism are both numerous and complex, fatty acid desaturases have been shown to play a key role in determining both plasma and tissue fatty acid profiles. Moreover, emerging evidence demonstrates that variation in fatty acid desaturase genes can modify whole-body lipid metabolism.

The aim of this review is to highlight the current state-of-knowledge regarding three fatty acid desaturases: stearoyl CoA desaturase 1 (*Scd-1*), fatty acid desaturase 1 (*Fads1*), and fatty acid desaturase 2 (*Fads2*). We will also discuss human studies that have begun to explore the genetic contribution underlying the inter-individual variability that exists with regards to desaturase activity. This review will demonstrate that fatty acid desaturases represent an important point of consideration for research aimed at preventing and treating various diseases through personalized dietary interventions.

## Stearoyl Coenzyme Desaturase-1: Background

Stearoyl Coenzyme Desaturase-1 (SCD-1) is the enzyme that catalyses the endogenous biosynthesis of MUFAs (i.e. palmitoleic acid, C16:1n-7; oleic acid, C18:1n-9) from *de novo *synthesized or dietary saturated fatty acids (SFA, i.e. palmitic acid, C16:0; stearic acid, C18:0) [19,20]. Moreover, SCD-1 has a specific affinity for two of the most abundant saturated fatty acids found in diet: palmitic and stearic acids. Palmitic acid is the major lipid in palm tree oils; however, it can also be found in other vegetable and animal sources. Stearic acid is found predominantly in fats and oils from animals and vegetables, and is usually consumed in meats, cocoa and processed shortening, spreads and baked products. The increased consumption of SFA-enriched foods characterizes Western dietary habits and is associated with the dramatic increases in cardiovascular disease and obesity [13,14]. As the rate-limiting enzyme responsible for catalyzing the conversion of SFAs into MUFAs, SCD-1 has become an interesting target in an attempt to understand the onset and development of the aforementioned diseases.

Rodent *Scd-1 *knockout models have been invaluable for advancing our understanding of SCD-1 function and regulation. While the goal of this review is not to provide a comprehensive description of SCD-1 biochemistry, it is nevertheless important to provide a brief overview of the current state-of-knowledge regarding this enzyme in order to appreciate the recent progress made in human-based research. A thorough overview of SCD-1 biochemistry can be obtained in reviews by Ntambi and colleagues [21,22]. Recent findings suggest that SCD-1 activity is tightly regulated, and that this regulation is disrupted in various disease states; however, it remains unclear whether perturbations in SCD-1 activity cause disease or are simply a response to disease. Furthermore, it appears that the degree of SCD-1 activity may underlie different health outcomes. Studies using *Scd-1 *knockout models revealed that an increase in SCD-1 activity is tightly associated with an obese phenotype, while a decrease in SCD-1 activity favours the development of cardiovascular complications due to a build-up of SFAs [23,24]. Whether alterations in SCD-1 activity induce these diseases is unknown; however, these associations are not isolated cases. Indeed, other disease states such as insulin resistance, metabolic syndrome, and cancer are also characterized by disturbances in SCD-1 activity [21,25-28]. These findings reinforce that SCD-1 is a critical player in the regulation of whole-body metabolism and is a promising target for therapeutic interventions [29].

To study the role of SCD-1, researchers have used rodents with either a naturally occurring *Scd-1 *deletion (asebia strain) or transgenic *Scd-1 *deletions. A lack of *Scd-1 *caused significant decreases in tissue triglycerides (TG), cholesterol esters (CE), wax esters, and diacylglycerols (DAG), as well as a reduction in lipid synthesis and an increase in β-oxidation, thermogenesis and insulin sensitivity, in the liver, muscle, and brown adipose tissue [19,20]. The resulting decrease in SCD-1 activity also led to a reduced desaturation index (i.e. the ratio of 18:1/18:0 and 16:1/16:0) [23,30]. Furthermore, *Scd-1 *deficient mice were found to be resistant to diet-induced obesity and characterized by decreased body weight, improved insulin sensitivity, and decreased hepatic steatosis [31,32].

While such data suggests a decrease in SCD-1 activity may be beneficial for weight management, emerging research indicates this reduction may also contribute to atherosclerosis; thus reinforcing that maintaining a balance in SCD-1 activity is paramount to optimize health. In 2008, MacDonald et al. studied the effect of *Scd-1 *deficiency on atherosclerosis in a hyperlipidemic, low-density lipoprotein receptor (LDLR)-deficient mouse model fed a Western diet [24]. LDLR-/- control mice developed diet-induced diabetes and obesity in the short term and atherosclerosis in the long term. When *Scd-1 *was additionally disrupted in a group of LDLR-/- mice, these animals exhibited lower body weights but increases in atherosclerotic lesion areas at the aortic root and plasma inflammatory markers (IL-6, ICAM-1, IL-1β and IL-12p70) [24]. This suggests that the ability of SCD-1 to metabolize an increased intake of dietary SFA is critical in order to prevent atherosclerosis. Further confirming the importance of balanced SCD-1 activity, Fessler et al. analyzed the influence of SCD-1 on inflammatory pathways by studying the associations between SFA, n-3 PUFAs, and toll-like receptor 4 (TLR4) - an activator of the innate immune system [33]. The authors demonstrated that the accumulation of SFA following *Scd-1 *deletion promoted the development of inflammation and disease via TLR4-mediated signalling pathways. Taken together, *Scd-1 *appears to play an important role in maintaining a balance in lipid profiles that, when deregulated, can contribute to inflammation, atherosclerosis, hypertriglyceridemia, and metabolic syndrome. While rodent research provides fundamental information regarding mechanism of action, the extent to which this knowledge can be translated to humans is still unknown.

## Interactions between diet and SCD-1

Few human intervention studies exploring the dietary regulation of SCD-1 are available to date. In 2002, Attie et al. analyzed the associations between diet, plasma TG, and SCD-1 activity in 429 healthy, Caucasian adults [23]. Participants were placed on a low-fat/high-carbohydrate diet (61-65% energy from carbohydrates) for 4-6 weeks. Changes in plasma lipids and lipoproteins levels were examined following the short-term intervention. This study revealed that before the dietary intervention, the C18:1/C18:0 desaturation ratio, an in vivo measure of SCD-1 activity, accounted for 11% of the variation in plasma triglyceride concentrations. However, after the consumption of a diet enriched in carbohydrates, the desaturation ratio accounted for over 40% of the variation in individuals whose triglyceride levels increased after the intervention. This suggests that SCD-1 may play a role in mediating carbohydrate-induced lipemia; therefore future research that analyzes SCD-1 activity within this context is warranted.

In 2004, Shiwaku et al. also analyzed the relationship between the 18:1/18:0 desaturation ratio and triglyceride levels [34]; however, the authors additionally explored the impact of ethnicity and dietary habits, assessed by the levels of plasma n-3 PUFA, on this relationship. The study recruited participants from three distinct ethnic groups: Japanese (n = 411), Korean (n = 418), and Mongolian (n = 251). Japanese participants consumed more fish than Koreans, who in turn consumed more than Mongolians. They found that fish consumption was positively correlated with plasma levels of n-3 PUFA. In line with the previously mentioned study by Attie and colleagues, significant associations were observed between the SCD-1 desaturation ratio (18:1/18:0) and plasma triglyceride levels in Japanese and Mongolians groups. Interestingly, this association was non-significant in the Korean group, suggesting ethnic differences. While Japanese and Koreans consume similar quantities of fish, differences in the degree to which SCD-1 activity affected triglyceride levels were observed; further reinforcing potential ethnic-specific factors that regulate plasma triglyceride levels above and beyond the influence of diet. When comparing the three ethnicities, the authors reported that the 18:1/18:0 desaturation ratio, n-3 PUFAs, age, gender, BMI, insulin resistance, and free fatty acids accounted for more than 50% of the variance in plasma triglyceride levels in Japanese and Mongolians individuals. In contrast, these same factors accounted for only 28% of plasma triglyceride varition in Koreans. Furthermore, hypertriglyceridemia was correlated with an increase in SCD-1 activity and decrease in plasma n-3 PUFA in an ethnic-specific manner. In Mongolian participants however, triglyceride levels were reduced independent of their low plasma n-3 PUFA concentrations, which the authors attributed to their low fish consumption. These findings suggest that SCD-1 activity is sensitive to diet and, more importantly, varies according to the genotype of distinct ethnic populations. Future nutrigenomic research should consider integrating the analysis of ethnic-specific variation in the *Scd-1 *gene, SCD-1 activity, and dietary habits in order to further clarify the role of this desaturase on clinical parameters commonly associated with metabolic diseases such as obesity and diabetes.

## Genetic Variation in Scd-1 and its Impact on Human Disease

Initial studies in subjects with familial combined hyperlipidemia (FCHL) revealed that this genetic condition is characterized by alterations in the lipid profile that may be explained in part by changes in SCD-1 activity [23,35]. While it does not appear that *Scd-1 *variants are responsible for FCHL, this does not preclude the notion that genetic variation in *Scd-1 *may affect enzyme activity and, subsequently, contribute to disease development.

Indeed, evidence suggests that genetic variation in *Scd-1 *may be associated with metabolic and physical parameters characterizing various disease states [36]. Furthermore, genetic studies suggest that these associations may be due to the effect of *Scd-1 *gene variants on SCD-1 activity, which consequently modifies plasma lipid profiles. Taken together, this reinforces the importance of unravelling the influence of genetic variation in *Scd-1 *on disease, especially when its interaction with dietary nutrients may modify disease development.

The first study examining genetic variation in *Scd-1 *and its impact on disease was published in 2004 [37]. Liew et al. analyzed the association between *Scd-1 *polymorphisms and susceptibility to type 2 diabetes in 608 diabetic and 600 control subjects of Irish and British descent. Despite the relatively small sample size used in this study, a borderline association was observed between the *rs41290540 *single nucleotide polymorphism (SNP) and diabetes risk (p = 0.059), in which the frequency of the minor allele was higher in the diabetic group [37]. However, this promising association was not replicated in an independent follow-up study performed by the same authors. While the lack of reproducibility is of concern, the results do not exclude the possibility that the minor allele for this *Scd-1 *SNP affects particular traits related to type 2 diabetes [37]. As such, future studies in larger cohorts may clarify the association between the *rs41290540 *SNP and diabetes risk. In 2007, Warensjo et al. analyzed the association between *Scd-1 *polymorphisms, SCD-1 activity, obesity, and insulin sensitivity in a population of 1143 elderly Swedish men [36]. They reported that the minor alleles of four *Scd-1 *SNPs *(rs10883463, rs7849, rs2167444 *and *rs508384) *were associated with a lower BMI, smaller waist circumference, and improved insulin sensitivity. For instance, the minor allele for *rs7849 *was correlated with a 23% increase in insulin sensitivity and 4 cm decrease in waist circumference. In many aspects, the phenotype associated with these minor alleles reflected the metabolic changes seen in *Scd-1 *deficient mice. Consequentially, the authors hypothesized that these minor alleles led to decreased SCD-1 activity; however, no significant change in SCD-1 activity was observed in individuals with these alleles. While it remains possible this is related to the relatively small cohort, it is more likely that measuring desaturation ratios in plasma fails to accurately represent long term or tissue-specific changes in SCD-1 activity [36].

## Fatty acid Desaturases: Background

The delta-6 desaturase (D6D) and delta-5 desaturase (D5D) are membrane-bound enzymes that catalyze the rate-limiting formation of long-chain PUFA [38,39]. These two enzymes are encoded by the fatty acid desaturase 2 (*Fads2*) and 1 (*Fads1*) genes, respectively. The *Fads1 *and *Fads2 *genes are found in a head-to-head fashion in a cluster on human chromosome 11 (11q12-q13.1), separated by an 11 kb region [38,40]. A third fatty acid desaturase gene (*Fads3*) also lies in the *Fads *cluster; however, the function of its translated product remains unknown [41]. Since all three *Fads *genes have 12 exons, 11 introns and share a common location in chromosome 11, it is believed that they arose evolutionarily from gene duplication and acquired substrate specificity over time [38].

**Figure 1:**
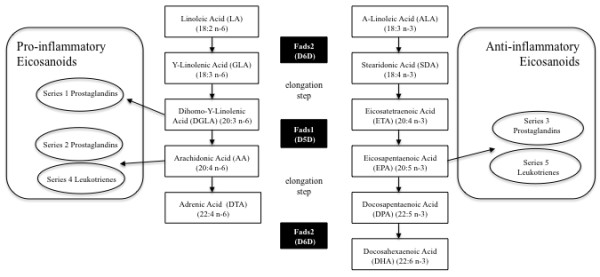
**Fatty acid desaturases in PUFA and eicosanoid biosynthesis**. The D6D (*Fads2*) and D5D (*Fads1*) enzymes play important roles in the biosynthesis of polyunsaturated fatty acids (PUFA). Moreover, these desaturases lead to the generation of pro-inflammatory (via n-6 PUFA) and anti-inflammatory (via n-3 PUFA) eicosanoids.

D6D catalyzes the conversion of α-linolenic acid (ALA, 18:3n-3) and linoleic acid (LA, 18:2n-6) into stearidonic acid (STD, 18:4n-3) and γ-linolenic acid (GLA, 18:3n-6), respectively (Figure 1). This is followed by an elongation step, after which D5D introduces a double bond at the Δ5 position in a 20-carbon fatty acid chain. D5D catalyzes the conversion of eicosatetraenoic acid (ETA, 20:4n-3) and dihomo-γ-linolenic acid (DGLA, 20:3n-6) into eicosapentaenoic acid (EPA, 20:5n-3) and arachidonic acid (AA, 20:4n-6), respectively [38,39] (Figure 1). The aforementioned PUFAs have important roles in maintaining membrane integrity and regulating cellular signaling events [5]. Irregularities in membrane fatty acid composition have been associated with several human diseases, such as Alzheimer's disease [42,43], atopic eczema [44], autoimmune diseases [2], cancer [45], and coronary heart diseases [3,46,47]. Furthermore, D6D and D5D activity is also known to regulate the levels of pro-inflammatory and anti-inflammatory eicosanoids derived from PUFAs [48]. The pro-inflammatory eicosanoids derived from AA are now known to contribute to the development of allergies [2,49], cardiovascular disease [50,51], and cancer [52]. Despite the wide-spread implications for *Fads *in the development of disease, only a few studies have directly studied the regulation of fatty acid desaturases in human tissue [53]. As such, the use of rodent models has provided important insight regarding the roles of these enzymes on lipid metabolism and disease.

The inhibition of D5D and D6D in an edema rodent model demonstrated that a marked decrease in inflammation was correlated with decreased levels of AA in liver, plasma and peritoneal cells [54]. Moreover, the inhibition of D6D was correlated with decreases in edema. These results strongly agree with other rodent studies which demonstrated that decreased levels of AA reduced the eicosanoid pool and attenuated AA-mediated signalling pathways regulating inflammatory responses [50,55]. In 2002, Hansen-Petrik et al. used two in vivo models of colorectal carcinogenesis (Apc^Min/1 ^mice and nude mice injected with HT-29 colonocytes) to examine the role of D6D on colon cancer [52]. As observed in other studies, the inhibition of D6D led to decreases in AA and an accumulation of its precursor LA. Furthermore, D6D inhibition reduced the number of tumours by 36-37% and decreased primary tumour size by 35%. When AA was directly supplemented, the effects of the D6D inhibitor on fatty acid composition and tumourigenesis in mice were abrogated. These results reinforce the importance of D6D activity in maintaining AA levels to regulate the production of inflammatory signalling molecules. Therefore, studying the extent by which genetic variation and dietary habits influence the regulation of D6D and D5D activity may lead to a better understanding of how these factors mediate the susceptibility to various diseases.

## The Role of Genetic Variation in *Fads1 *and *Fads2 *in humans

Emerging research has demonstrated that genetic variation in *Fads1 *and *Fads2 *are associated with alterations in fatty acid composition that may subsequently modify an individual's propensity for disease. Although such research has only recently begun, significant associations have already been identified between *Fads *polymorphisms and fatty acid levels, which are summarized in Table 1.

**Table 1:** Summary of SNP-trait associations identified for *Fads1 *and *Fads2*

dbSNP ID	Gene, Region	Allele (M/m)	Population Size & Ethnicity	Association findings	Ref
***Fads1***					

*rs174537*	*Fads1 *intron	G/T	n = 1453, Caucasian (ITA) n = 1120, Caucasian (USA)	GG < GT&TT: ↑LA, ALA; ↓EDA, AA, EPA, LDL-C & total cholesterol in serum	[61]

*rs174544*	*Fads1 *3' UTR	C/A	n = 727, Caucasian (GER)	CC < CA < AA: ↑LA, EDA, DGLA & ALA; ↓GLA, AA, DTA, EPA & DPA in serum	[49]

*rs174546*	*Fads1 *3' UTR	C/T	n = 1144, Caucasian (EUR)	CC < CT < TT: ↑LA, DGLA, ALA; ↓AA, EPA, DHA in serum CC < CT&TT: ↓GLA in serum	[60]

*rs174545*	*Fads1 *3' UTR	C/G	n = 876, Caucasian (ITA)	CC < CG < GG: ↑AA, AA/LA, EPA/ALA; ↓LA, ALA in RBC	[51]
			
			n = 658, Caucasian (ITA)	CC < CG < GG: ↑AA; ↓ALA, EDA in serum and RBC	[57]

*rs174547*	*Fads1 *intron	T/C	n = 1809, Caucasian (GER)	TT < TC < CC: ↑PC aa C36:3/PC aa C36:4	[65]
			
			n = 422, Caucasian (UK)	TT < TC < CC: ↑PC aa C36:3/PC aa C36:4	[65]

*rs174553*	*Fads1 *intron	A/G	n = 727, Caucasian (GER)	AA < AG < GG: ↑LA, EDA, DGLA & ALA; ↓GLA, AA, DTA, EPA & DPA in serum	[49]
			
			n = 69, 74% Caucasian (CAN)	AA < AG&GG: ↑LA; ↓AA in plasma and RBC during gestation	[68]
			
			n = 54, 74% Caucasian (CAN)	AA < AG < GG: ↑EDA; ↓AA, EPA, DPA in breast milk	[68]

*rs174556*	*Fads1 *intron	C/T	n = 727, Caucasian (GER)	CC < CT < TT: ↑LA, EDA, DGLA & ALA in serum; ↓GLA, AA, DTA, EPA & DPA in serum	[49]
			
			n = 658, Caucasian (ITA)	CC < CT < TT: ↑EDA in serum & RBC; ↓AA in serum & RBC	[57]

*rs174561*	*Fads1 *intron CpG-island, promoter region	T/C	n = 727, Caucasian (GER)	TT < TC < CC: ↑LA, EDA, DGLA, ALA in serum; ↓GLA, AA, DTA, EPA & DPA in serum	[49]
			
			n = 658, Caucasian (ITA)	TT < TC < CC: ↑EDA in serum & RBC; ↓AA in serum & RBC	[57]
			
			n = 309, Caucasian (DUT)	TT < TC < CC: ↑GA, EA LA, EDA, DGLA; ↓GLA, AA, DTA, DHA in plasma phospholipids TT < TC < CC: ↑DGLA; ↓AA, DTA, EPA, DHA in breast milk	[67]

***Fads2***					

*rs174570*	*Fads2 *intron	C/T	n = 727, Caucasian (GER)	CC < CT&TT: ↑EDA in serum; ↓GLA, AA, DTA in serum	[49]
			
			n = 876, Caucasian (ITA)	CC < CT < TT: ↑LA, ALA in RBC; ↓AA, AA/LA in RBC	[51]
			
			n = 658, Caucasian (ITA)	CC < CT < TT: ↑LA in RBC, ↓AA in serum & RBC	[57]
			
			n = 1144, Caucasian (EUR)	CC < CT < TT: ↑LA, ALA; ↓AA in serum CC < CT&TT: ↓GLA in serum	[60]

*rs174572*	*Fads2 *intron	C/T	n = 1144, Caucasian (EUR)	CC < CT < TT: ↑LA, DGLA, ALA; ↓AA, EPA in serum CC < CT&TT: ↓GLA, DHA in serum	[60]

*rs174575*	*Fads2 *intron	C/G	n = 54, 74% Caucasian (CAN)	CC < CG&GG: ↓AA, EPA, DPA & DHA in breast milk	[68]
			
			n = 309, Caucasian (DUT)	CC < CG < GG: ↑GA, LA, EDA, DGLA; ↓AA, DTA, DHA in plasma phospholipids CC < CG < GG: ↑DGLA; ↓PEA, AA, DTA, EPA, DPA in breast milk	[67]

*rs174583*	*Fads2 *intron	C/T	n = 727, Caucasian (GER)	CC < CT < TT: ↑LA, EDA, DGLA & ALA in serum; ↓GLA, AA, DTA, EPA & DPA in serum	[49]
			
			n = 876, Caucasian (ITA)	CC < CT < TT: ↑LA, ALA in RBC; ↓AA, AA/LA, EPA/ALA in RBC	[51]
			
			n = 658, Caucasian (ITA)	CC < CT < TT: ↑ALA, EDA, LA in serum & RBC; ↓AA in serum & RBC	[57]

*rs174589*	*Fads2 *exon/intron boundary	G/C	n = 727, Caucasian (GER)	GG < GC < CC: ↑LA, EDA & DGLA in serum; ↓GLA, AA, EPA & DPA in serum	[49]
			
			n = 876, Caucasian (ITA)	GG < GC&CC: ↑AA, AA/LA in RBC; ↓LA, EPA/ALA in RBC	[51]
			
			n = 658, Caucasian (ITA)	GG < GC < CC: ↑AA, in serum & RBC, ↓EDA in serum	[57]
			
			n = 1144, Caucasian (EUR)	GG < GC < CC: ↑LA, DGLA, ALA; ↓AA, EPA in serum GG < GC&CC: ↓GLA in serum	[60]

*rs174602*	*Fads2 *exon/intron boundary	A/G	n = 727, Caucasian (GER)	AA < AG < GG: ↑LA, EDA in serum; ↓AA in serum	[49]
			
			n = 1144, Caucasian (EUR)	AA < AG < GG: ↑LA, ALA; ↓AA, EPA in serum	[60]

*rs174611*	*Fads2 *intron	T/C	n = 876, Caucasian (ITA)	TT < TC < CC: ↑LA, ALA in RBC; ↓AA, AA/LA, EPA/ALA in RBC	[51]
			
			n = 658, Caucasian (ITA)	TT < TC < CC: ↑EDA in serum ↓AA in serum & RBC	[57]
			
			n = 1144, Caucasian (EUR)	TT < TC < CC: ↑LA, DGLA, ALA; ↓AA in serum TT < TC&CC: ↓GLA, EPA in serum	[60]

*rs174616*	*Fads2 *intron	G/A	n = 1144, Caucasian (EUR)	GG < GA < AA: ↑LA, DGLA; ↓AA in serum GG < GA&AA: ↑ALA; ↓EPA, DHA in serum	[60]

*rs174620*	*Fads2 *intron	T/C	n = 727, Caucasian (GER)	TT < TC < CC: ↑DGLA in serum; ↓AA in serum	[49]

*rs2072114*	*Fads2 *exon/ intron boundary	A/G	n = 727, Caucasian (GER)	AA < AG&GG: ↑ LA, EDA in serum; ↓GLA, AA, DTA in serum	[49]
			
			n = 1144, Caucasian (EUR)	AA < AG < GG: ↑LA, DGLA, ALA; ↓AA in serum AA < AG&GG: ↓GLA in serum	[60]

*rs2524299*	*Fads2 *intron	A/T	n = 876, Caucasian (ITA)	AA < AT < TT: ↑LA in RBC; ↓AA, AA/LA in RBC	[51]
			
			n = 658, Caucasian (ITA)	AA < AT < TT: ↓AA in serum and RBC	[57]

*rs482548*	*Fads2 *3' UTR	C/T	n = 727, Caucasian (GER)	CC < CT&TT: ↑AA, DTA in serum	[49]

*rs498793*	*Fads2* intron	G/A	n = 658, Caucasian (ITA)	No significant association found with lipid species in serum or RBC	[57]
			
			n = 1144, Caucasian (EUR)	GG < GA < AA: ↑AA; ↓LA in serum GG < GA&AA: ↑EPA in serum	[60]

*rs526126*	*Fads2 *exon/ intron boundary	G/C	n = 727, Caucasian (GER)	GG < GC < CC: ↑DGLA in serum	[49]
			
			n = 1144, Caucasian (EUR)	GG < GC < CC: ↑LA, ALA; ↓AA in serum	[60]

*rs968567*	*Fads2 *5'UTR	C/T	n = 1144, Caucasian (EUR)	CC < CT < TT: ↑LA, DGLA, ALA; ↓AA, EPA CC < CT&TT: ↓GLA in serum	[60]

*rs99780*	*Fads2* intron	C/T	n = 727, Caucasian (GER)	CC < CT < TT: ↑levels of LA, EDA, DGLA, ALA in serum; ↓GLA, AA, EPA, DPA in serum	[49]

***Intergenic***					

*rs174627*	*Fads2/Fads3 *Intergenic	C/T	n = 876, Caucasian (ITA)	CC < CT < TT: ↑LA, ALA in RBC; ↓AA, AA/LA, EPA/ALA in RBC	[51]
			
			n = 658, Caucasian (ITA)	CC < CT < TT: ↑EDA, AA in serum	[57]

*rs17831757*	*Fads2/Fads3* Intergenic	T/C	n = 658, Caucasian (ITA)	TT < TC&CC: ↓AA in serum	[57]

*rs3834458*	*Fads1/Fads2 * intergenic, CpG-island	T/del*	n = 727, Caucasian (GER)	TT < T/del < del/del: ↑LA, EDA, DGLA, ALA in serum; ↓GLA, AA, DTA, EPA, DPA in serum	[49]
			
			n = 1694 (case), Caucasian (ITAL)	TT < T/del < del/del: ↑ETE & EDA in adipose tissue; ↑ plasma TG; ↓EPA, GLA & AA in adipose tissue	[58]
			
			n = 876, Caucasian (ITA)	TT < T/del & del/del: ↑LA, ALA; ↓AA, AA/LA, EPA/ALA in RBC	[51]
			
			n = 658, Caucasian (ITA)	TT < T/del < del/del: ↑ALA, EDA in serum; ↑LA, EDA in RBC; ↓AA in serum and RBC	[57]
			
			n = 309, Caucasian (DUT)	TT < T/del < del/del: ↑GA, EA, LA, EDA, DGLA; ↓GLA, AA, DTA, DHA in plasma phospholipids TT < T/del < del/del: ↑LA, DGLA; ↓AA, DTA, EPA, DPA, DHA in breast milk	[67]

*rs968567*	*Fads1/Fads2 *intergenic, CpG-island, promoter region	G/A	n = 727, Caucasian (GER)	GG < GA&AA: ↑LA, DGLA in serum; ↓GLA, AA, EPA & DPA in serum	[49]

Several SNPs have been examined in *Fads1 *and *Fads2*; however, only significant associations (*p *< 0.05) are listed in this table. Associations between *Fads *genes and several fatty acids have been identified, including: palmitelaidic acid (PEA, C16:1n-7), gadoleic acid (GA, C20:1n-9), erucic acid (EA, C22:1n-9), linoleic acid (LA, C18:2n-6), γ-linoleic acid (GLA, C18:3n-6), eicosadienoic acid (EDA, C20:2n-6), dihomo-γ-linolenic acid (DGLA, C20:3n-6), arachidonic acid (AA, C20:4n-6), adrenic acid (DTA, C22:4n-6), α-linolenic acid (ALA, C18:3n-3), eicosatrienoic acid (ETE, 20:3n-3), eicosapentaenoic acid (EPA, C20:5n-3), docosapentaenoic acid (DPA, C22:5n-3), docosahexaenoic acid (DHA, C22:6n-3), phosphatidylcholine diacyl C36:3 (PC aa C36:3), and phosphatidylcholine diacyl C36:4 (PC aa C36:4). Other abbreviations: LDL-C, low-density lipoprotein cholesterol; TG, triglyceride; RBC, red blood cell; M/m, major and minor allele; and *del, deletion. A ↑ indicates an increase and a ↓ represents a decrease.

In 2006, Schaeffer et al. analyzed the associations between genetic variants in human *Fads1 *and *Fads2*, and fatty acid composition in serum phospholipids [49]. Eighteen SNPs located in the *Fads *cluster were analyzed in a cohort of 727 Caucasian adults from Germany. The authors identified several SNPs, as well as statistically reconstructed haplotypes in the *Fads *cluster, that were strongly associated with changes in phospholipid fatty acid composition. For the most affected fatty acid, AA, up to 28% of its variance could be explained by the SNPs in the studied cluster, followed by its precursors eicosadienoic acid (EDA, C20:2 n-6) at 12% and DGLA at 10%. It was observed that subjects carrying various minor alleles had higher levels of LA and ALA, and decreased levels of AA and EPA in serum phospholipids. Furthermore, a haplotype analysis reinforced that carriers of minor alleles had increased levels of ALA and LA, and decreased levels of AA and EPA. The authors suggested that individuals with these minor alleles may have a lower prevalence for immunological diseases, such as allergic rhinitis and atopic eczema due to the significantly lower concentrations of AA observed; however, this remains to be demonstrated fully by future research since significance was lost after correcting for multiple testing.

A subsequent study verified these associations in an independent cohort composed of 535 participants from the Bavarian Nutrition Survey II (BVS-II) [56]. Rzehak and colleagues conducted a haplotype analysis on fatty acid composition in both serum phospholipid and red blood cell (RBC) membranes. The results confirmed that minor allele haplotypes were strongly linked to changes in fatty acid composition, such as decreased AA level in circulating serum phospholipids. Furthermore, minor allele haplotypes were found to influence fatty acid composition at a cellular level, which was represented by fatty acid alterations in the RBC membrane.

Two other studies have assessed fatty acid composition in both serum phospholipids and RBC membranes with the purpose of measuring short-term transient alterations and long-term chronic modifications in fatty acid status, respectively. Malerba and colleagues examined the association between *Fads *gene variants and fatty acid composition in 658 cardiovascular disease patients recruited in the Verona Heart Project [57]. This study revealed that homozygote and heterozygote carriers of various minor alleles had a fatty acid profile characterized by significantly lower levels of AA in both serum phospholipids and erythrocyte membranes, as found independently in two other studies [49,56]. After multiple test adjustments, significance diminished, and the only significant association that remained was that of a constructed haplotype within the *Fads *cluster and the levels of AA in serum and RBC membranes [57]. Martinelli et al. examined the same SNPs used in the previous study; however, they included an association analysis between *Fads *gene variants and coronary artery disease (CAD), as well as desaturase activity in the RBC membranes of 610 CAD and 266 CAD-free subjects from the Verona Heart Project [51]. Almost all SNPs studied in the *Fads *cluster were associated with the desaturation ratios for AA/LA and EPA/ALA, but no single variant was significantly associated with CAD or CAD-free subjects. However, haplotypes with a greater number of risk alleles were more frequent in CAD patients than CAD-free individuals, as well as being associated with a higher desaturation ratio (AA/LA), and increases in high sensitivity C-reactive protein (hs-CRP), a common inflammatory marker. Regression analyses adjusted for multiple testing revealed that the AA/LA ratio is indeed a significant predictor of CAD. The authors concluded that individual *Fads1 *and *Fads2 *polymorphisms had little to no effect on CAD risk; however, an additive model of risk alleles, which corresponds to a higher desaturase activity, was more frequent in CAD subjects and showed a significant effect on CAD susceptibility [51].

Additional studies have explored the relationship between diet and *Fads*, and the influence on cardiovascular disease. In 2007, Baylin et al. analyzed the effect of a common deletion variant in the *Fads2 *promoter (*rs3834458*) on ALA concentrations in adipose tissue and the risk of nonfatal myocardial infarction (MI) in a Costa Rican population of men and women diagnosed as survivors of a first acute MI [58]. The authors reported that the heterozygous deletion variant of the *Fads2 *promoter was prevalent in almost half of the population (48%) and was associated with a decrease in serum EPA, GLA, and AA and an increase in eicosatrienoic acid (ETE, C20:3 n-3) and EDA, as well as TG, with increasing number of copies of the variant allele. However, this deletion was not significantly associated with MI, nor did it attenuate the association between ALA in adipose tissue and MI [58]. In contrast with the author's original hypothesis, reduced activity of D6D did not translate into decreased protection from MI as a result of reduced conversion of ALA into its very-long-chain products. However, Baylin et al. suggested that the results of this study may have been masked by the high availability of dietary ALA compensating for the defective transcription of D6D in individuals with the deletion variant. This research involving the *Fads2 *promoter SNP (*rs3834458) *was followed up by Truong et al., who studied the effect of genetic variation in the *Fads2 *SNP on the association between ALA and the prevalence of the metabolic syndrome. The studied cohort consisted of 656 metabolic syndrome subjects and 1159 metabolic syndrome-free subjects from a Costa Rican population-based case-control study examining heart disease [59]. A significant trend was observed for a lower prevalence ratio (PR) of metabolic syndrome in individuals with high concentrations of adipose tissue ALA, compared to individuals with low adipose tissue ALA. Moreover, a lower PR was associated with higher levels of adipose tissue ALA in homozygote and heterozygote carriers of the common T-allele. This trend, however, lost significance in homozygote carriers of the deletion allele with high levels of adipose tissue ALA. This suggests that in individuals with the *Fads2 *deletion allele, the high consumption of ALA may only have an attenuated beneficial effect on the prevalence of the metabolic syndrome, which demonstrates the influence of genetic variation in *Fads2 *on the mediation of disease risk. Nevertheless, the extent to which genetics mediates the association between diet and disease needs to be explored in further studies.

Most association studies to date have focused on analyzing the influence of genetic variation in the *Fads *cluster in adult subjects; however, analyzing this influence in younger subjects provides an alternate perspective for understanding how genetic variation affects lipid metabolism. Adolescents have been exposed to fewer environmental challenges than adults, thus the influence of the genetic makeup on the inter-individual phenotypic variability is more direct in an adolescent cohort. Bokor et al. recently examined the relationship between *Fads *SNPs, plasma fatty acids, TGs and desaturase activity in a cohort of European adolescents [60]. The results revealed similar links to those found in adults, in that significant associations were observed between minor alleles of several *Fads *SNPs, and various fatty acids, TGs, and D6D and D5D activity in plasma. In agreement with previous adult studies, a significant increase in LA and decrease in AA and D5D activity in plasma were associated with minor alleles in the *Fads *cluster. Moreover, the associations observed were highly significant, which can be attributed to the lack of confounding factors masking the effects of genetic variability on the phenotype. Further research is necessary to elucidate the full impact of these genetic effects, and recent evidence suggests studying younger cohorts will provide additional insight.

Two genome-wide association studies (GWAS) have recently confirmed the importance of *Fads *genes on lipid metabolism and quantitative traits associated with disease. Tanaka et al. conducted a GWAS in 1075 participants from the InCHIANTI study in order to identify gene variants that may explain variability in plasma PUFA levels [61]. The authors found a significant association between a SNP in *Fads1 *(*rs174537*) and plasma levels of AA that accounted for 18.6% of the variance in AA levels. Carriers of the minor allele had lower levels of AA, EDA, and EPA, and higher levels of LA and ALA in plasma; suggesting a decrease in D5D activity. Furthermore, these individuals had lower levels of total cholesterol and low-density lipoproteins, indicating that this minor allele may favour a plasma lipid profile that decreases the risk for cardiovascular disease. These findings were subsequently validated by the authors in a second study cohort [61]. These reproducible findings suggests that genetic variation in *Fads1 *may not only explain differences in plasma lipid profiles between individuals, but may also influence the risk for cardiovascular disease. Moreover, such studies may shed further light on the wide disparity in conversion efficiency of ALA to docosahexaenoic acid (DHA, C22:6 n-3) observed between individuals, which can range from < 1% to as much as 10% [3,62-64]. In 2010, Illig et al. conducted a large GWAS that identified strong relationships between traits associated with the metabolic syndrome and CVD, and several genetic variants. Serum from 1809 adults from a German population study (KORA) and 422 female adults from a British population study (TwinsUK) were measured, and the concentrations of 163 metabolic traits were analyzed [65]. The strongest association observed was between a SNP in the *Fads1 *gene (*rs174547*) and the ratio of product (phosphatidylcholine diacyl C36:4) to precursor (phosphatidylcholine diacyl C36:3) fatty acids in both study cohorts. The authors demonstrate that considering metabolites as phenotypic traits, combined with the power of a GWAS, is an effective approach for the identification of new candidate SNPs. Furthermore, they revealed that the use of metabolite concentration ratios as a surrogate measurement of enzymatic activity reduced variation and yielded strong statistical associations with a very high degree of significance. Hence, future studies wishing to discover new genetic variants associated with disease risk should consider integrating genetic and metabolomic approaches in order to identify more robust associations.

These studies establish the importance of the *Fads *genes on the regulation of risk factors associated with health and disease, and as such, demonstrate the need for future research that elucidates both the molecular and physiological impact of polymorphisms in the *Fads. *Moreover, the observed influence of genetic variation on whole-body lipid metabolism positions *Fads *as intriguing candidates for future nutrigenomics research.

## Genetic Variation in *Fads *and Breastfeeding

Interesting evidence suggests that the influence of genetic variation in *Fads *on circulating and tissue fatty acid profiles, which contribute to modifying risk factors for the development of disease, may have a trans-generational effect [66-68]. Indeed, previous research has focused on analyzing these effects within an individual (i.e. a single generation); however, recent studies have demonstrated that the dietary habits of gestating and/or lactating mothers may also impact their offspring.

For example, Xie and Innis examined how genetic variants in *Fads1 *and *Fads2 *may affect lipid composition in gestating women as well as their breast milk during lactation [68]. This study analyzed six SNPs and the results demonstrated a direct correlation between genetic variants and lipid composition in plasma phospholipids, RBC membranes and breast milk. Homozygotic carriers of the minor allele for the *rs174553 *SNP had lower AA and higher LA in plasma phospholipids and RBC membranes, and a lower D6D and D5D product to precursor ratio at 16 and 36 weeks of gestation. Individuals with these minor alleles also had significantly lower AA, EPA and docosapentaenoic acid (DPA, C22:5 n-3), but higher EDA, in breast milk. Since levels of fatty acids in the embryo and newborn baby are directly associated to maternal fatty acid levels (via placental transfer during gestation and breast milk consumption after birth), any variation in the maternal intake of fatty acids whose levels in blood and tissue are sensitive to genetic variability may prove critical for fetal growth and development [66]. Along the same avenue of research, Caspi et al. analyzed the influence of genetic variation in *Fads2 *on the association between breastfeeding and infant IQ [66]. Breastfeeding exposes babies to increased concentrations of maternal DHA and AA, crucial fatty acids known to enhance cognitive development. Therefore, it was hypothesized that breastfeeding may contribute to a higher IQ after adjustment for multiple confounders, such as social class, maternal IQ, genotypic effects on exposure to breastfeeding and genotypic differences in intrauterine growth. The authors reported that the *Fads2 *SNP (*rs174575*) interacted with breastfeeding to influence IQ levels in the two cohorts studied: the Dunedin cohort (1037 Caucasian children from New Zealand), and the E-risk cohort (2232 British infant twins). For both cohorts, it was observed that breastfed children carrying the common C-allele highly benefitted from breastfeeding, compared to children who were not fed breast milk. On the other hand, there was no effect of breastfeeding on IQ in GG homozygotes. These results further support the notion that the maternal diet plays a key role in the development of cognitive function and that, importantly, genetic variation in *Fads2 *can alter this association. Indeed, the results reveal that lipid desaturases are critical in the process of cognitive development and that the interaction between breastfeeding (i.e. maternal dietary habits) and variation in these genes could potentially influence and explain the observed differences in IQ. These findings suggest that genetic research should not overlook the influence of environmental factors on heritability.

Expanding on the diet-gene effects observed in pregnant mothers, a recent study by Moltó-Puigmartí et al. analyzed the influence of *Fads *polymorphisms on the association between fish intake and DHA levels in maternal plasma and breast milk [67]. The study cohort consisted of 309 Dutch pregnant women from the KOALA Birth Cohort Study. With the use of a food frequency questionnaire, fish and fish oil intake were estimated and associated to plasma and breast milk fatty acid levels measured during gestation and 1 month postpartum, respectively. Furthermore, the effects of genetic variation in 3 SNPs in the *Fads *cluster (*rs174561, rs174575, rs3834458) *were analyzed in order to study the relationship between fish oil intake and DHA concentrations in plasma and breast milk. The results showed that the association between genotype and fatty acid levels in plasma and milk were additive and that DHA levels increased in plasma and breast milk in accordance to the number of major alleles. Interestingly, it was observed that increased fish and fish-oil intake elevated the concentration of DHA in plasma, irrespective of genotype; however, in breast milk, DHA concentrations only increased in carriers of the major alleles. These results demonstrate that modifying the transfer of DHA from mother to child through dietary interventions will vary based on the mother's genotype. Further studies are needed to identify the mechanisms by which genetic variation in the *Fads *genes modulate the levels of DHA in breast milk and the eventual impact of this genetic variation on the offspring.

## Future Directions

It is widely recognized that perturbations in lipid metabolism are associated with the development of human disease. Moreover, the regulation of lipid metabolism is truly a complex systems biology paradigm that involves genes and proteins in multiple tissues throughout the organism. Consequently, it is crucial to analyze the regulation of these molecules using comprehensive techniques such as GWAS and 'omics-based research. The application of 'omics-based research provides a complementary and innovative approach to improve our understanding of the role of desaturases in human metabolism, as recently exemplified by Tanaka et al. and Illig et al. [61,65]. A few studies have demonstrated that interactions between diet and gene variants mediate the risk of chronic disease [58,59]. Indeed, the genetic makeup of an individual may modulate, to an extent, the association between nutritional intake and clinical parameters linked to disease. Further research in this promising avenue of exploration should try to elucidate the extent to which these interactions influence the inter-individual difference for disease risk and try to identify candidate SNPs that may be used as biomarkers for diagnosis and personalized therapeutic treatment.

Furthermore, given that dietary habits are tightly linked to disease susceptibility, it is possible that dietary habits have confounded the significance of previous studies, especially when one considers that the consumption of specific fatty acids may mask any changes in desaturase activities that are genetically determined. Several of the studies outlined in this review have demonstrated that differences related to genotype are masked by the consumption of ALA, EPA and/or DHA [34,58]. Consequently, proper adjustment for nutritional intake is of paramount importance when examining associations that will eventually provide relevant data for the implementation of dietary interventions that aim at preventing and managing disease.

The study by Shiwaku and colleagues has further reinforced the complex relationship between genes and diet by demonstrating that ethnicity is another important covariate to consider in gene-association studies using multi-ethnic populations. This issue may be mute when establishing cohorts from isolated populations; however, this becomes extremely relevant when establishing cohorts from multi-ethnic urban centres. Ethnicity can be considered a combination of lifestyle, diet, and gene differences; however, these important factors are often overlooked in studies involving multi-ethnic populations. The significant impact of lifestyle on genetic diversity was recently illustrated by Novembre et al, who reported that individuals from across Europe can be geographically clustered using 500,000 SNPs [69]. While this may not apply to any one SNP in particular, it clearly reinforces the important interactions between lifestyle and genes (i.e. lifestyle genomics) within a population. In addition, it is difficult to assess the influence of immigration in study populations, which may modify the association results. Novembre et al. suggested that an individual's genetic make-up can be used to infer their geographic origin [69]. Therefore one can ask, for example, at what point does a Caucasian European who has immigrated to North America become a Caucasian American at the genetic level? It is entirely possible that immigrants never fully adapt genetically to their new environmental surroundings, meaning this is a considerable challenge to overcome in order to identify reproducible gene variants that modify disease risk. To further reinforce this notion, we used the HapMap database to extract all SNPs located in a region containing *Scd-1 *± 10 kb from the four ethnic groups: 1) CEU - Utah residents with European ancestry, 2) CHB - Han Chinese from Beijing China, 3) JPT - Japanese from Tokyo Japan, and 4) YRI - Yoruba from Ibadan Nigeria (Table 2). It is immediately apparent that the frequency of common variants differs between the populations. The CHB and JPT populations are in close proximity from a geographic perspective, and this is reflected by their highly similar genetic makeup when compared to the other two populations. While the differences illustrated with this example may seem intuitive, it reinforces that SNPs that are associated with a particular trait in one population may not be relevant in other populations simply because they occur only infrequently. Therefore, considerable effort to homogenize a study cohort must be taken prior to performing association studies in order to account for potential lifestyle and ethnic confounders.

**Table 2:** Single nucleotide polymorphisms (SNPs) in the Stearoyl-CoA desaturase (*Scd*-1) gene in four ethnic populations

CEU	CHB	JPT	YRI
**rs2060792**	**rs2060792**	**rs2060792**	

	*rs7088953*		*rs7088953*

*rs17669878*	*rs17669878*	*rs17669878*	*rs17669878*

*rs11190478*	*rs11190478*	*rs11190478*	*rs11190478*

*rs735877*	*rs735877*	*rs735877*	*rs735877*

*rs11599710*	*rs11599710*	*rs11599710*	*rs11599710*

*rs670213*	*rs670213*	*rs670213*	*rs670213*

	*rs640773*	*rs640773*	*rs640773*

	*rs639060*	*rs639060*	*rs639060*

**rs1502593**	**rs1502593**	**rs1502593**	**rs1502593**

	*rs612472*	*rs612472*	*rs612472*

	*rs529271*	*rs529271*	*rs529271*

**rs522951**	**rs522951**	**rs522951**	**rs522951**

	*rs681278*	*rs681278*	*rs681278*

*rs11190480*	*rs11190480*	*rs11190480*	*rs11190480*

*rs11190483*	*rs11190483*	*rs11190483*	

			**rs7904902**

*rs3870747*	*rs3870747*	*rs3870747*	*rs3870747*

**rs3071**	**rs3071**	**rs3071**	

*rs3829160*	*rs3829160*	*rs3829160*	

*rs3793766*	*rs3793766*	*rs3793766*	*rs3793766*

			**rs12247426**

*rs3793767*	*rs3793767*	*rs3793767*	*rs3793767*

*rs3793768*	*rs3793768*	*rs3793768*	*rs3793768*

*rs2234970*	*rs2234970*	*rs2234970*	*rs2234970*

	*rs599961*	*rs599961*	*rs599961*

**rs10883463**			

**rs3978768**	**rs3978768**	**rs3978768**	

*rs11557927*	*rs11557927*	*rs11557927*	*rs11557927*

**rs10883465**	**rs10883465**	**rs10883465**	**rs10883465**

*rs7849*	*rs7849*	*rs7849*	*rs7849*

	*rs560792*	*rs560792*	*rs560792*

*rs508384*	*rs508384*	*rs508384*	*rs508384*

	*rs539480*	*rs539480*	

*rs1393491*	*rs1393491*	*rs1393491*	*rs1393491*

**rs1393492**	**rs1393492**	**rs1393492**	**rs1393492**

**rs575338**			

*rs490726*	*rs490726*	*rs490726*	

**rs11190488**			

**rs569184**			

*rs569910*	*rs569910*	*rs569910*	*rs569910*

	*rs570844*	*rs570844*	*rs570844*

	*rs608622*	*rs608622*	*rs608622*

			*rs17113027*

This table highlights the ethnic differences in *Scd-1 *gene variation, using data from the International HapMap Project database (HapMap Data Rel/24phaseII Nov08, on NCBI B36 assembly, dbSNP b126). Data from the 4 populations was extracted: 1) CEU: CEPH- Utah residents with European ancestry, 2) CHB: Han Chinese in Beijing, China, 3) JPT: Japanese in Tokyo, Japan, and 4) YRI: Yoruba in Ibadan, Nigeria. Tag SNPs (tSNPs; in bold font) were consistently selected with Haploview software V4.1 using a minor allele frequency (MAF) > 5% and r^2^≥0.8.

## Conclusion

Evidence now exists demonstrating that genetic variation in *Scd-1*, *Fads1*, and *Fads2 *can modify desaturase activity. Initial studies support the notion that genetic variation in these genes affects the onset and development of various diseases characterized by perturbations in lipid metabolism. Furthermore, studies that analyze the interactions between genes and diet have begun to highlight the influence that maternal dietary habits may have on their offspring's growth and development, and long-term disease risk factors. Therefore, a nutrigenomics approach will prove beneficial for unravelling the interactions between diet and desaturase genes in ethnically distinct populations. Such research will contribute to the development of tailored dietary strategies for the prevention and control of disease.

## Competing interests

The authors declare that they have no competing interests.

## Authors' contributions

All authors contributed to the writing of this manuscript and have approved its final version.
